# A Devil's dictionary for mental health

**DOI:** 10.1192/pb.bp.116.055442

**Published:** 2017-10

**Authors:** Philip Timms

**Affiliations:** 1 King's College London

## Abstract

Clinical psychiatry, for all its emphasis on scientific rigour, is mediated mainly by words rather than by numbers. As with other professional areas, it has developed its own set of jargon words and phrases. Many of these are not the technical terms traditionally seen as jargon, but standard English words and phrases used in an idiosyncratic way. They therefore go unnoticed as jargon, while enfeebling our communications. I have used the template of Ambrose Bierce's *The Devil's Dictionary* to highlight some examples, with the aim of helping us all to talk, write and, perhaps, think more clearly.

A review of health literacy suggests that patients often do not understand what doctors are trying to tell them – and, rather obviously, tells us that we should be more straightforward.^1^ But it is not just us. The US boss of Pfizer has exasperated a British parliamentary committee with his obscure business jargon.^2^ Farrington has highlighted the chasm between clinical and managerial language.^3^ Brian Lask pilloried the ‘infestation’ of family therapy publications with jargon, pomposity and impenetrability.^4^ And, although they were mainly addressing technical obfuscation, a pair of psychologists have trenchantly characterised much of the research communication in their field as ‘bullshit’.^5^ They recommend translating ‘others' procedures and outcomes in(to) ordinary language’ to separate out any discernible content from the chaff of jargon.

But I am not here going to consider how we talk to patients or managers – or even researchers and academics. I have been thinking about how we talk to each other. It can be said that technical jargon does not matter inside a profession because each participant understands the words involved. It only becomes a problem when a professional tries to communicate with outsiders.^6^ But I think we may have a problem in day-to-day communication between those of us involved in mental health services. Over the past few years I have noticed the appearance of words and phrases which, because of their novelty and lack of definition, create real problems with communication. Many of these can be seen as ‘weasel’ words or phrases. They carry a covert meaning or overtones of meaning – or, sometimes, no meaning at all. They can be defined as ‘something that someone says either to avoid answering a question clearly or to make someone believe something that is not true’ (http://dictionary.cambridge.org/dictionary/english/weasel-words). Some are more technical, but all can be seen as forms of jargon owing to their characteristic use in medicine and psychiatry.

## What is jargon?

But what is jargon? The current version of the online Oxford Dictionary defines it as ‘Special words or expressions used by a profession or group that are difficult for others to understand’. It also tells us that it been with us for nearly 400 years, being derived from Late Middle English, originally in the sense of ‘twittering, chattering’, later ‘gibberish’ (www.oxforddictionaries.com/definition/english/jargon). Earlier dictionaries are more robust: ‘Barbarous or debased language: gibberish: speech full of technical terms etc’.^7^ Synonyms include slang, cant, idiom, argot, patter and gobbledegook (www.thesaurus.com/browse/jargon). Nobody much likes jargon, even though we all seem driven to use it.

At first hearing, such language may not sound at all technical. The words or phrases are drawn from day-to-day English, but, as we use them, their meaning starts to dissolve. They become the aforementioned weasel words because they are either novel or of broad, inclusive meaning. Those inside a profession find that they can use such language in an idiosyncratic way to preserve professional authority and power.^8^ If you cannot understand a discussion, you cannot participate. The young may feel that they have to use this language to be an authentic part of professional life. The old, consciously or unconsciously, realise that it can be used to conceal, confuse, stonewall and neuter objections or criticism. Moreover, jargon is mostly categorical and conceptual, not concrete and specific. It can present an intimidating facade behind which, if one takes the trouble to look, there is little of substance. Although unsavoury, is it not a victimless crime? Not according to the House of Commons Public Administration Select Committee. They felt that ‘the use of inaccurate, confusing or misleading official language which results in tangible harm, such as preventing individuals from receiving benefits or public services, should be regarded as maladministration’ (p. 16).^6^ Perhaps, then, we should take this issue more seriously.

One might, in an uncharitable moment, single out managers as the main offenders. However, I have heard colleagues from our sister professions talking impenetrably, as well as my fellow psychiatrists and, regrettably, myself. We are all guilty. So, I decided to round up some words and phrases that seem, to me, to be misused in this way. Some of these are specific to psychiatry and mental health, some are used across medicine. Some remain a local coinage, others have attained a more universal currency. We hear some in the front line, in wards and community teams, and others in committee rooms and consultation papers. Ambrose Bierce was a 19th-century journalist who was similarly intrigued by the ways in which language can be misused. I have used the template of his *The Devil's Dictionary*^9^ to produce my own, somewhat scattershot, rogue's gallery.

## My Devil's Dictionary

**Acting out**: an analytical term provoked by extravagant behaviour. Example: ‘the patient acted out by shouting in the lounge’. We are thereby relieved from the burden of considering the matter further.

**Adherence** (to medication, advice, etc.): a delusion, held by all doctors, most strongly by enthusiastic pharmacologists. For us, a mainstay of treatment. For our patients, an irritation best neglected. They are reluctant to reveal their disinterest, not wishing to distress their psychiatrist.

**Acuity**: a snappy euphemism which removes to arms-length the stress and risk of our under-resourced and risky services. Essential to reduce the anxiety felt in higher echelons of management.

**Behavioural**: a term used by those of us with no behavioural training, to describe behaviour of which we disapprove (*see* ‘Acting out’). Commonly combined with ‘just’ – ‘It's just behavioural’. Usually paired with the helpful conclusion: ‘he/she is not for our service’.

**Best evidence**: the cracked and misty lens with which the one-eyed man navigates the country of the blind.

**Care pathway**: a sequence of ideal interventions (*see* ‘Best evidence’) for a model patient. Lengthy and complicated, so best illustrated on a sheet of A3 paper. The dogged patient may be said to trudge, march or run the gauntlet on such a trek.

**Challenge**: the presentation of the impossible and implausible as a bold plan. Example: the NHS Challenge – save £20 billion in the next 3 years while improving quality. You can then, subsequent to the inevitable failure of the plan, pillory those involved as being old-fashioned, inflexible, not trying hard enough … according to taste.

**Clinician**: a drone, a harmless drudge who sees patients. They can apparently perform audit, supervision and research simultaneously (*see* ‘Job description’). Their ability to be in two places at once (*see* ‘Job description’) suggests that they would have been burned as witches in less scrupulously rational times.

**Consultation**: the practice of gathering views about a proposed change in a service. To complain that such views are subsequently ignored would be both harsh and naive (*see* ‘Listening event’ and ‘Efficiency saving’).

**Cover**: an illusion of doubles. It suggests that an overworked colleague will also do your work in your absence. Essential to maintain the appearance of an adequately staffed service.

**Cut**: the Voldemort word that must never, ever, be uttered (*see* ‘Efficiency saving’). Sometimes linked with ‘Transformation’.

**Distress**: a catch-all for every experience from mild anxiety to raging psychosis. As one gently minimises the more extreme and intractable varieties of experience, one can slyly insinuate that psychiatry is just a way to medicalise normal experience.

**Efficiency saving**: as the wolf to Red Riding Hood's grandmother. Cloaked in two benign words with which none can take issue, it stalks our services – and suddenly they disappear. *See also* ‘Challenge’ and ‘Cut’.

**Evidence**: the single academic paper that supports one's proposed course of action.

**Holistic**: a synonym for comprehensive, originating from the word ‘whole’. But where did the ‘W’ go? The spelling generates a spurious overtone of spirituality. Clearly not intentional; evidence of unconscious processes at work.

**Hypothesis**: a simple idea that leaves port to the sound of marching bands, but then founders on the rocks of bureaucracy or upon the craggy island of sober reflection.

**Integration**: the unicorn of service provision. Often spoken of but rarely seen. Some say they have seen it in Birmingham. Others say it is a mirage, glimpsed by the desperate.

**Innovation**: a novelty that attracts money.

**Inappropriate** (behaviour): rational behaviour of which we disapprove. Studiously neutral, it is commonly used in close proximity to an emphatic font. Example: ‘patients must *not* use our accident and emergency department inappropriately’.

**Job description**: a work of fantasy that masquerades as a workaday agenda. As nectar to the bee, it attracts naive applicants to your service while obscuring the inquisitive gaze of our College. Any subsequent disaffection can be met with the phrase ‘caveat emptor’.

**Just**: an excellent way to deny complexity. One can avoid the trouble of biopsychosocial formulation without admitting to idleness, ignorance or indifference. Example: ‘It's just… behavioural/social/drug-induced …’.

**Liaison**: the assurance that someone will, at some time, communicate something to someone. Example: ‘Community mental health team to liaise with primary care’. A common parasite of care plans and strategy documents. The phrase ‘liaison psychiatry’ can be honourably exempted.

**Listening event**: a talking shop.

**Metric**: a swaggering and self-confident synonym for ‘number’, ‘figure’ or ‘statistic’. It ballasts with false weight the inadequate and corrupted data that leak out of our struggling services.

**Medical model**: a synonym for the crudely biological. Essential if one has a sketchy acquaintance with medicine but a sincere desire to damn psychiatry. Best bolstered by a quote from an antique textbook which few have ever read – or even heard of.

**Mission statement**: a haiku of the obvious. A way for those remote from the front line to sincerely avow the commitments of an organisation. Only misfits and malcontents could assert that such statements are banal and platitudinous.

**New ways of working**: the promise that clinical drones (see above) can become queen bees. The admirable and novel element is that psychiatrists should, as far as possible, steer well clear of patients.

**Ongoing**: a way to communicate to the naive reader an impression of dogged and ceaseless activity.

**Paradigm shift**: a bit of a change. It proclaims how different (and how much better) your pet idea (*see* ‘Hypothesis’) is from everything that has gone before. Such shifts promise seismic change but most, mysteriously, register zero on the Richter scale of life.

**Prioritisation**: cutting one service to provide another. One service dies and another is born. Disney's circle of life.

**Quality indicator**: an easily measurable irrelevance.

**Quality improvement**: an unarguable good. It marvellously exempts management from any responsibility for the lack of armaments or ammunition and ‘empowers’ frontline platoons to sort out the subsequent carnage.

**Research**: the selection of an unrepresentative group of people, the provision of an unsustainable intervention and the careful selection of a rating cut-off point to show your intervention to its best advantage.

**Recovery**: a two-edged sword. To Tweedledum, an essential reorientation of services to patient priorities. To Tweedledee, a pretext for culling rehabilitation services.

**Reconfiguration**: another attempt to rearrange the chairs on the deck of the Titanic (*see* ‘Transformation’).

**Significance** (statistical) (*see also* ‘Research’): a simple number that lends colossal weight to negligible differences.

**Signposting**: a respectable way to rebuff those seeking help from our service. We direct the patient towards another step on their therapeutic pilgrimage (*see* ‘Care pathway’), braced by the pious assurance that another will meet their need. Much loved by hard-pressed commissioners for whom distance from our services is by far the best medicine.

**Target**: a worthy aim, applauded by all. The resources marshalled to meet it hyperperfuse privileged parts of the organisation but induce gangrene and necrosis elsewhere.

**Triage**: a battlefield technique to sift the doomed from the salvageable. Applied to civilians in peacetime when the money runs out.

**Transformation**: an ‘abracadabra’ word, the promise to turn an ugly duckling into a swan. This powerful spell can reduce resistance to modish technology or to shedding staff Memories mysteriously fail when the new swan proves to be just another maladroit fowl.

**Vision(s)**: in a patient, evidence of brain dysfunction. In ourselves, evidence of foresight, imagination and understanding.

**Work-life balance** (*see* ‘Job description’): an excellent way to communicate an uncomfortable truth. Example: ‘We know you need to work X+1 sessions to do the job, but we will only pay you for X sessions’. Dissimulation is charmingly paired with an expression of concern for one's welfare.

## Casting out demons

The American edition of the online Oxford Dictionary, somewhat uncharitably, states that weasel words are ‘intentionally ambiguous or misleading’.^7^ I am less judgemental and would suggest that they have a less deceitful function. They help us to feel more at ease with difficult truths we have both to confront ourselves and to present to others. However, although we may feel more comfortable, we will not be communicating as well as we could – and not thinking as clearly as we should. I would not go as far as the Local Government Association which was widely reported to have published, for its members, a list of banned words and phrases.^10^ After all, context is everything and, in spite of my accusations, some of these words may be used quite helpfully from time to time. But an awareness of them can serve us as the canary once served the coal miner: as a sign that something may not be quite right, and that we need to keep our wits about us. To the charge that I am a cynic, I confess that I do not have the stomach for it. I still shrink from the uncomfortable and cling to desperate and unreasonable hopes. I have been unable to yet become that paragon described inimitably by Bierce^9^ (p. 34) as ‘A blackguard whose faulty vision sees things as they are, not as they ought to be’.

This list is not comprehensive and is certainly not static. New weasel words will emerge as others wither and perish. Each of us can identify our own offenders. To pay attention to how we talk (and write) is not self-indulgent. It can help us to think more clearly, to communicate more meaningfully and to engage with reality rather than self-serving fantasy.
